# Mental Health of Psychologists During a Period of Cumulative Crises in Lebanon: The Predictive Role of Self-Esteem

**DOI:** 10.3390/healthcare14010080

**Published:** 2025-12-29

**Authors:** Rabab Bou Debs, Rudy S. Younes, Stephanie Abboud, Sandra Akoury, Jana Hamzeh, Joya Arab, Christina Mechref, Nadine Zalaket

**Affiliations:** 1 Department of Psychology and Social Sciences, Faculty of Arts and Sciences, Holy Spirit University of Kaslik (USEK), Mount Lebanon, Jounieh P.O. Box 446, Lebanon; stephanie.c.abboud@net.usek.edu.lb (S.A.); sandra.r.akoury@net.usek.edu.lb (S.A.); jana.h.hamzeh@net.usek.edu.lb (J.H.); joya.r.arab@net.usek.edu.lb (J.A.); christina.c.mechref@net.usek.edu.lb (C.M.); nadinezalaket@usek.edu.lb (N.Z.); 2 IDEES Multidisciplinary Research Group, Faculty of Arts and Sciences, Holy Spirit University of Kaslik (USEK), Mount Lebanon, Jounieh P.O. Box 446, Lebanon; rudy.s.younes@net.usek.edu.lb

**Keywords:** clinical psychologists, psychotherapists, educational psychologists, mental health, self-esteem, economic crisis

## Abstract

**Background/Objectives**: Since October 2019, Lebanon has faced continuous sociopolitical and economic instability. Clinical psychologists have played a central role in responding to rising mental health needs, yet little is known about their own psychological well-being. **Methods**: This study examined mental health outcomes among 157 certified psychologists (clinical and educational psychologists) working in Lebanon. A cross-sectional study was conducted with psychologists aged 30–53 years across all Lebanese governorates, who were recruited through snowball and word-of-mouth sampling. Participants completed validated measures of depression (PHQ-9), anxiety (LAS-10), perceived stress (PSS-10), subjective well-being (WHO-5), eating attitudes (EAT-26), and self-esteem (A-SISE). **Results**: Results showed that 44% of participants reported at least mild depressive symptoms, 14% met criteria for anxiety, and 57% experienced moderate to high perceived stress, while most showed no risk for eating disorders. Bivariate and multivariate analyses identified self-esteem as a predictive factor, negatively associated with depression, anxiety, and stress, and positively associated with subjective well-being. Additional risk factors included younger age, being unmarried, not having children, prior psychological history, health problems, lower income, and working as an educational rather than clinical psychologist. **Conclusions**: These findings highlight aspects of vulnerability among psychologists and underline the need for targeted interventions for at-risk groups. Strengthening self-esteem may contribute to enhancing clinicians’ mental health. However, these conclusions should be interpreted in light of several limitations, including the small sample size, the non-probability and gender-skewed nature of the sample, partly due to the relatively limited number of practicing psychologists in Lebanon.

## 1. Introduction

Clinical psychologists play a central role in the assessment, treatment, and prevention of mental health difficulties [1,2]. They work to enhance individuals’ emotional, cognitive, and relational functioning by providing psychotherapy, counseling, psychological evaluation, and psychoeducation [3,4]. However, their role implies substantial emotional labor. Indeed, psychologists routinely encounter clients’ distress, trauma, and suffering, which can contribute to professional stress, burnout, and emotional fatigue if not buffered by adequate support and personal coping resources [5,6]. The psychological well-being of clinicians themselves is crucial, not only for their own functioning, but for the quality and continuity of mental health services they provide [7].

The importance of clinicians’ mental health becomes even more pronounced in crisis and conflict-affected contexts. In such settings, psychologists frequently encounter acute and chronic trauma, heightened levels of distress, and increased demands for psychological support [8,9]. Research in conflict settings shows that mental health professionals are at elevated risk of experiencing distress and mental health concerns, as they are exposed to instability and widespread human suffering [10]. Clinical psychologists therefore serve dual roles: they are both providers of support to others and individuals personally embedded in the same socio-political conditions that generate distress. This dual exposure may amplify vulnerability to mental health difficulties and make understanding their well-being particularly important.

In recent years, Lebanon has been characterized by prolonged and cumulative crises that have led to unusual strain on mental health professionals. Since October 2019, the country has faced continuous sociopolitical and economic instability. Recurrent political unrest, governance failures, and infrastructure deterioration have contributed to widespread insecurity [11]. The financial collapse of 2019, described as one of the worst economic crises globally in recent history, drove nearly 78% of the population into poverty [12] and produced record inflation rates exceeding 250% [13]. These difficulties were compounded by the COVID-19 pandemic, which intensified social and economic hardship [14,15]. In August 2020, the Beirut Port explosion, deemed one of the most powerful non-nuclear explosions recorded, produced widespread psychological trauma, grief, and loss [16,17]. More recently, the war in 2023–2024 contributed to displacement, fear, and renewed instability [18,19]. These cumulative crises have destabilized daily life and profoundly affected mental health needs across the country [20].

Yet, while the population’s mental health needs have increased [20], psychologists themselves also live within the same conditions of uncertainty and collective trauma. This parallel exposure raises key questions about clinicians’ own psychological well-being, resilience, and the factors that may heighten their vulnerability. Although extensive research has examined mental health outcomes among the Lebanese population more broadly, far less is known about the well-being of those providing mental healthcare. To our knowledge, no research has given attention to the well-being of mental health professionals themselves, despite their central role in societal recovery and support. Understanding the mental health status of psychologists is, therefore, a public health priority.

In this context, clinical psychologists have played a central role in responding to rising mental health needs, yet they themselves are embedded in the same conditions of uncertainty and collective distress. While research in Lebanon has documented elevated rates of depression, anxiety, and mental illness, to our knowledge, no studies have explored the mental health of clinical psychologists. In fact, existing research in Lebanon has largely focused on the general population or specific age groups such as young adults [19,21]; however, the psychological well-being of psychologists themselves has yet to be examined. Given the unprecedented cumulation of crises, it is essential to assess how clinical psychologists in Lebanon are coping psychologically. This study seeks to fill a gap in the literature by examining the mental health profile of certified psychologists working in Lebanon, focusing on several dimensions of mental health, namely depression, anxiety, perceived stress, subjective well-being (SWB), and eating disorders (EDs). Depression and anxiety are common markers of psychological distress, perceived stress reflects cumulative strain related to work and broader circumstances, and subjective well-being captures overall psychological functioning and life satisfaction. EDs, although less frequently examined in mental health research, were also included, as emerging evidence from Lebanon indicates that war-related stress and lower quality of life may increase vulnerability to disordered eating, making this mental health outcome potentially relevant [22,23].

Moreover, self-esteem has been widely recognized as a core psychological resource that buffers against distress [24,25]. Indeed, self-esteem is closely tied to resilience and adaptive coping, which enables individuals to maintain a sense of psychological stability when facing adversity [26,27]. From a self-concept perspective, higher self-esteem provides a more coherent and positive sense of self, which facilitates effective coping and adaptation [28]. Likewise, vulnerability–stress models propose that low self-esteem heightens sensitivity to stressors and increases the likelihood of psychological distress [29]. For clinicians who routinely work with trauma and emotional suffering, self-esteem may therefore play a crucial role in sustaining coping capacity and overall psychological well-being. Yet, its role among psychologists practicing in a prolonged crisis context has not been adequately examined. Therefore, the study has the following aims:

1. Assess the prevalence of depression, anxiety, stress, subjective well-being, and eating-related attitudes among psychologists in Lebanon;
2. Examine the association between socio-demographic/professional variables and mental health outcomes;
3. Evaluate the predictive role of self-esteem in mitigating psychological distress among psychologists in Lebanon.

## 2. Materials and Methods

### 2.1. Study Design and Procedure

This study adopted a cross-sectional design and was conducted between June and July 2025. The participants were certified psychologists who are currently working in Lebanon. The participants were distributed across all Lebanese governorates in Lebanon: Akkar, Baalbek, Beirut, Mount Lebanon, North Lebanon, South Lebanon, Bekaa, and Nabatiyeh.

We collected our sample size by using the snowball and word-of-mouth techniques [30,31]. We distributed our questionnaire by Google Forms across social networks such as WhatsApp, Instagram, and Facebook. While a census was attempted by contacting psychologists in Lebanon through the Order of Psychologists in Lebanon (LOPsy), not all invited individuals responded. Google Forms’ setting that prevents respondents from submitting more than one entry was used to prevent any duplicate responses. The final dataset reflects those who completed the survey. Moreover, we reassured the participants about the anonymity of the questionnaire during the process of data collection. Therefore, the confidentiality of the study was maintained. We specifically explained the objective of our study. Furthermore, participants had the liberty of enrolling in this study without pressure and did not receive financial benefits for their participation. The study protocol was approved by the ethical committee of the Higher Center for Research (HCR) of the Holy Spirit University of Kaslik (USEK), under protocol number 2025-010.

### 2.2. Sample Size and Participants

Using G*power software version 3.1.97 (based on linear multiple regression R^2^ deviation from zero), the minimal required sample size was calculated as 135 participants, based on a theoretical f^2^ of 0.15 (medium effect size), an alpha error of 0.05, a beta error of 20%, and 14 covariates to be included in the final model.

Psychologists, male and female, aged at least 30, were included in the study to ensure that participants had a minimum level of professional experience or training. This would allow for the assessment of mental health among psychologists with some established practice or training. Thus, psychologists who refused to participate in the survey, those who were not in Lebanon, those not certified, and those under 30 were excluded from this study.

At the time of this study, there were 829 licensed psychologists at the Lebanese Order of Psychologists in Lebanon (LOPsy). With 157 participants included, the study reached approximately 19% of the national psychologist population, and likely a higher proportion among those aged over 30, although the exact percentage of those above 30 is difficult to determine. This sample size meets the minimal statistical power requirements (higher than 135) and represents a substantial segment of psychologists in Lebanon. Nevertheless, the relatively small sample should be taken into account when interpreting the generalizability of the findings at the national level.

### 2.3. Instruments

The data were collected through a questionnaire composed of two parts. The first collected sociodemographic data, and the second part comprised the standardized scales measuring several aspects of mental health.

#### 2.3.1. Patient Health Questionnaire Depression Scale (PHQ-9)

PHQ-9 is a brief scale based on the diagnostic criteria for depression according to the DSM-IV and allows for the screening of depression symptoms and their severity by a simple self-test [32,33]. This scale includes 9 items using a 4-point Likert scale (0—‘not at all’; 3—‘almost every day’ and a final item with answers ranging from ‘not difficult at all’ to ‘extremely difficult.’ As a standard, and according to a validation study conducted in Lebanon, it categorizes depression severity as 0 to 4 (none), 5 to 9 (mild), 10 to 14 (moderate), 15 to 19 (moderately severe), and 20 to 27 (severe) [32,34]. The PHQ-9 scale was translated and validated to suit the Lebanese population [34,35,36]. In this study, alpha Cronbach was 0.813 for the internal consistency test.

#### 2.3.2. Lebanese Anxiety Scale (LAS-10)

LAS-10 is an anxiety screening scale that was specifically developed for the Lebanese population. It consists of 10 items in total that use a 5-point Likert (0—‘there is no’; 4—‘very strong’). The scores obtained by each item are added to provide a final score, allowing for the interpretation of the level of anxiety, which worsens with the increase in the final score [37]. Previous studies have used a mean cutoff score of 13.5 [21]. In this study, the alpha Cronbach was 0.875 for the internal consistency test.

#### 2.3.3. Perceived Stress Scale (PSS-10)

The PSS-10 [38] is a widely used instrument designed to assess the degree to which individuals perceive situations in their lives as stressful. It consists of 10 items rated on a 5-point Likert scale ranging from 0 (“never”) to 4 (“very often”), with four positively stated items (4, 5, 7, and 8) being reverse-scored. The total score, obtained by summing all items, ranges from 0 to 40, with higher scores indicating greater perceived stress. Scores between 0 and 13 reflect low stress, 14–26 moderate stress, and 27–40 high stress [39,40]. The PSS-10 has demonstrated good internal consistency, with a Cronbach’s alpha of approximately 0.85 [41]. The scale has been validated in a Lebanese sample [42]. In this study, alpha Cronbach was 0.837 for the internal consistency test.

#### 2.3.4. WHO-5 Well-Being Index

Subjective well-being was measured using the WHO-5 Well-Being Index. It is a brief, five-item questionnaire from the World Health Organization that measures subjective mental well-being over the past two weeks. It asks about feelings of “cheerful and in good spirits,” “calm and relaxed,” “feeling active,” “woke up fresh and rested,” and being “filled with things that interest me” on a 6-point scale from “at no time” to “all of the time.” The scale has been validated across multiple countries worldwide [43], and has been validated in Arabic across numerous countries, including Lebanon [44]. In this study, the alpha Cronbach was 0.902 for the internal consistency test.

#### 2.3.5. The Eating Attitudes Test (EAT-26)

EAT-26 is used to identify the presence of “eating disorder risk” based on attitudes, feelings, and behaviors related to eating. There are 26 self-report questions assessing general eating behavior and five additional questions assessing risky behaviors [45]. The EAT-26 can aid in the screening and diagnosis of eating disorders such as anorexia nervosa, bulimia nervosa, and binge eating disorder. EAT-26 has been validated in a Lebanese sample [46]. A score of 20 or higher on the EAT-26 indicates a high level of concern [45]. In this study, the alpha Cronbach was 0.883 for the internal consistency test.

#### 2.3.6. The Arabic Single-Item Self-Esteem Scale (A-SISE)

The Single-Item Self-Esteem Scale is a widely used one-item questionnaire used to measure global self-esteem, using a 5-point Likert scale where participants rate how true a statement is of them [47]. It is a valid and reliable alternative to longer scales. Its Arabic version has been validated in a Lebanese sample [48].

### 2.4. Data Analysis

Data analysis was performed using SPSS software version 27. The normality of the distribution of the mental health variables was confirmed by calculating the skewness and kurtosis; skewness and kurtosis values between −1 and +1 are considered acceptable to prove a normal univariate distribution [49,50]. In addition, Q-Q plots were inspected (see Figures S1–S6), showing that observed quantiles closely followed the theoretical normal distribution with only minor deviations at the tails. These results jointly indicate acceptable univariate normality for the variables analyzed.

Student’s *t*-test was used to compare two means, ANOVA test to compare three or more means, and Pearson’s test to correlate two continuous variables.

Then, linear regressions were conducted, taking the mental health outcomes as dependent variables in each. In line with methodological guidelines and previous studies, the factors that showed a *p* < 0.25 in the bivariate analysis as independent variables were considered for the regression models, using a ‘purposeful selection of variables’ at the screening stage to avoid excluding potentially important covariates [51,52,53,54,55]. It should be noted that the *p* < 0.25 criterion was used only as a screening rule for candidate covariates, not as a formal criterion for statistical significance in the final interpretation of results. In the linear regressions, multicollinearity was assessed using the Variance Inflation Factor (VIF), with a threshold of VIF > 5 considered indicative of problematic correlation among predictors [56]. VIF ranged between 1.05 and 1.37, indicating no concerning multicollinearity among predictors.

Significance was considered for a *p*-value < 0.05. For analyses involving multiple comparisons, *p*-values were adjusted using the Bonferroni–Holm correction [57], applied separately for each mental health outcome, to reduce Type I error.

## 3. Results

A total of 157 participants completed the questionnaire, with 87.9% females and 84.7% having a master’s degree. All participants’ characteristics can be found in Table 1.

**Figure 1:**
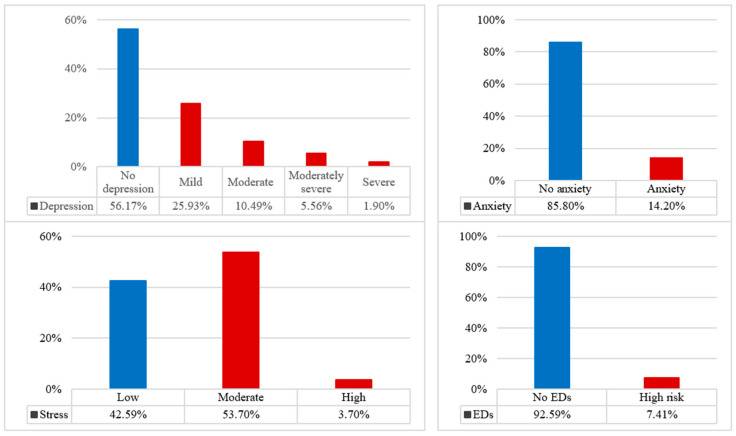
Levels of mental health outcomes (depression, anxiety, stress, and EDs) (N = 157). Note: The thresholds reported for each variable correspond to the established cutoff scores provided by the respective psychometric scales mentioned in Section 2.

Regarding mental health status, approximately 44% of participants showed at least mild depression based on the PHQ-9 scores, while 14% met the threshold for anxiety based on the LAS-10. More than half of the sample (57%) experienced moderate to high levels of perceived stress according to the PSS-10 scores, though severe stress was rare. The vast majority of participants showed no risk of eating disorders (Figure 1).

Bivariate analyses revealed several significant associations between demographic variables and mental health outcomes (Table 2). Age was significantly associated with anxiety levels. Marital status showed significant associations with depression, anxiety, and stress. Participants with children reported lower levels of depression and anxiety compared to those without. Psychological history was significantly associated with all mental health variables except eating disorders. Health problems were linked to higher depression and anxiety. Monthly salary demonstrated significant associations with anxiety and stress levels. Educational psychologists reported higher depression and stress compared to clinical psychologists. Work shift was associated with depression and subjective well-being, though interpretation requires caution given the small number of night shift workers.

Then, the bivariate correlations between self-esteem and the mental health dimensions were conducted. Depression, anxiety, and stress showed significant negative correlations with self-esteem, while SWB indicated a significant negative correlation. EDs showed no association with self-esteem (Table 3).

A multivariate linear regression analysis was conducted to examine the independent contribution of self-esteem to each mental health outcome while controlling for relevant demographic variables (Table 4). Self-esteem emerged as a significant predictor across all models, demonstrating negative associations with depression, anxiety, and perceived stress, and a positive association with subjective well-being. The models explained substantial variance in mental health outcomes, with R^2^ values ranging from 0.388 to 0.449, indicating that self-esteem accounts for a considerable proportion of the variance in psychological well-being among this sample of Lebanese psychologists. The complete multivariate analysis, including the models and the effect of socio-demographic variables, is provided in the Supplementary Materials (Tables S1–S4).

## 4. Discussion

This cross-sectional study examined the mental health status of certified psychologists working in Lebanon and explored the role of self-esteem as a predictive factor. The study assessed depression, anxiety, perceived stress, subjective well-being, and eating disorder risk among 157 psychologists across all Lebanese governorates during a period of ongoing national crises.

The findings revealed a mixed mental health profile among the sample. Approximately 44% of participants reported at least mild depressive symptoms, 14% met the threshold for anxiety, and 57% experienced moderate to high levels of perceived stress. However, severe stress was relatively rare, subjective well-being scores were moderate, and eating disorder risk was minimal across the sample. Overall, these rates suggest relatively better mental health outcomes compared to the general Lebanese population, which has been documented to experience significantly elevated rates of psychological distress amid the country’s compounding crises [19,21,58]. When compared to available data from the general Lebanese population using the same instruments, approximately three-quarters met criteria for anxiety on the LAS-10, and 95% reported moderate or high stress on the PSS-10. Two studies examined depression in the general population using the PHQ-9, with one showing that almost half of adults and the other showing that most scored at least mild depression. These comparisons suggest that psychologists experience relatively lower distress than the general population [19,21,58]. This effect may be attributable to several profession-specific factors, including psychological literacy, access to therapeutic resources, familiarity with mental health management strategies, and the resilience developed through professional training and practice [59]. Nevertheless, the presence of nearly half of our sample reporting depressive symptoms and over half experiencing elevated stress indicates that substantial proportions of psychologists remain at risk and warrant targeted support.

An important finding of this study is the suggested predictive role of self-esteem across multiple mental health dimensions. Self-esteem demonstrated significant negative associations with depression, anxiety, and perceived stress, and a positive association with subjective well-being, accounting for substantial variance in these outcomes. These findings align with established literature, worldwide and in Lebanon, positioning self-esteem as a fundamental psychological resource that buffers against distress and promotes adaptive functioning [24,25,60,61,62]. This pattern is consistent with self-concept theory and vulnerability–stress models, which propose that a coherent and positive sense of self enhances coping capacity, whereas low self-esteem heightens sensitivity to stressors and increases risk for psychological distress [28,29]. For psychologists navigating the demanding nature of their profession within a crisis-affected context, maintaining healthy self-esteem appears to be an important factor in sustaining psychological well-being.

This study identified several demographic and professional factors associated with heightened mental health vulnerability among psychologists. For starters, age emerged as a significant predictor of anxiety, with younger psychologists reporting higher anxiety levels. This finding aligns with the worldwide literature suggesting that younger age is associated with higher anxiety [63]. However, it should be noted that age did not correlate with the other mental health outcomes, which is not congruent with research supporting that mental illnesses, like depression and eating disorders, are more prevalent with younger age [64]. This inconsistent pattern should be interpreted cautiously, as the effects were weak and may reflect limited small subgroup sizes. It is also possible that age-related differences in depression, stress, subjective well-being, or eating-related attitudes exist in the broader population of psychologists but did not appear here due to the small subgroups. Therefore, the observed trend for anxiety should be considered preliminary rather than conclusive.

Interestingly, years of professional experience showed no significant association with mental health outcomes, a finding that diverges from a portion of the literature suggesting experience-related adaptation to distress [65,66,67]. This may indicate that contextual factors in Lebanon’s current crisis exceed the typical protective effects of experience [68]. However, another explanation can be provided by recent studies, which have shown that a relationship exists between years of experience and distress among healthcare professionals, but it is not a linear one. According to this research, in the first few years of work, there is an initial decrease in exhaustion levels as work years increase. At approximately 4–8 years of work experience, exhaustion begins to rise, reaching its peak. Subsequently, with further increases in work years, exhaustion continued to decline until reaching a minimum later on in one’s career [65,69]. However, this type of relationship could not be reliably measured in this study, given the relatively small sample size.

Marital status and parenthood showed associations with several mental health outcomes at the bivariate level, with married psychologists and those with children reporting lower depression, anxiety, and stress. However, after applying appropriate corrections and adjusting for multiple variables, these associations were no longer significant. This pattern is inconsistent with previous studies suggesting that marital support and parental roles provide emotional resources and a sense of meaning that protect against distress [70,71,72]. In the present sample, these demographic factors do not appear to exert an independent influence on mental health; rather, their initial associations are likely accounted for by other variables included in the multivariate analyses. As such, these findings should be interpreted cautiously, and future studies with larger and more diverse samples are needed to clarify whether marital and parental status meaningfully contribute to psychologists’ well-being in the Lebanese context.

It was expected that psychologists with a personal history of psychological disorders would report higher levels of distress. However, in both the bivariate and adjusted models, this association was significant only for depression. This may be because depressive disorders are often long-lasting and can become chronic, especially after several episodes. As a result, people may develop residual symptoms or enduring cognitive-emotional vulnerabilities, such as persistent negative affectivity, low mood, fatigue, and sleep issues, among others [73,74]. These residual features make depression more likely to show lasting associations with prior psychological history. In contrast, the other measured mental health outcomes, especially perceived stress and subjective well-being, tend to be more context-dependent and shaped by situational demands rather than by long-lasting clinical traits. This may explain why prior psychological history did not independently predict these domains once other variables were considered. Accordingly, while personal psychological history remains an important indicator of vulnerability, its effects in this sample appear to be specific to depressive symptoms rather than uniform across all mental health outcomes.

Economic factors did not show a clear or consistent association with the mental health outcomes examined. This suggests that salary alone may not meaningfully account for psychologists’ mental health. This absence of a robust association contrasts with population-level evidence linking financial strain to poorer psychological outcomes [21,75]. One plausible explanation is that profession-related stressors, such as high caseloads and emotional burden, may outweigh the effect of salary and, therefore, dilute its predictive value. Additionally, other individual factors, such as self-esteem, showed stronger associations with mental health outcomes, potentially absorbing the variance that financial variables might otherwise explain. Future research should investigate a broader range of financial indicators, including financial well-being or subjective financial security, particularly given previous findings in Lebanon showing the buffering role of financial well-being for mental health [21,75].

Professional specialization demonstrated a possible and marginal association with depression. In the bivariate analyses, educational psychologists showed higher depression scores than clinical psychologists. However, this difference did not remain when the correction for multiple comparisons was applied. In the multivariable regression (see Table S1), depression showed a significant association with professional specialization level after taking into account other covariates, but this effect should be interpreted cautiously given its borderline significance and sensitivity to analytic approach. Taken together, the findings suggest that professional specialization may be related to depressive symptoms, yet the evidence is not robust and appears to diminish under more conservative correction procedures. This finding seems consistent with evidence that the workplace setting is frequently associated with depressive symptoms [76,77]. It can be explained by enduring work-related dynamics that differ between educational and clinical settings, such as limited autonomy and sustained role strain. Additionally, educational psychologists may have fewer opportunities for structured supervision or clinical support, potentially amplifying vulnerability to depressive symptoms. For other mental health outcomes, there were no meaningful differences between clinical and educational psychologists.

### 4.1. Limitations

Despite the novelty of this study, several limitations should be acknowledged when interpreting these findings. First, the study relied exclusively on self-report measures, which are inherently vulnerable to recall bias, social desirability bias, and under-reporting of symptoms [78]. These concerns may be accentuated in a sample of mental health professionals who are most likely familiar with psychological constructs and screening tools, potentially influencing how they appraise or report their own mental states. This may have partially inflated model estimates and may have contributed to the relatively high effect sizes observed in the associations between self-esteem and mental health outcomes.

Second, although self-esteem emerged as a central variable in the analysis, it was assessed using a single-item scale. While single-item scales are often as reliable and valid as larger ones [79], it should be acknowledged that they may have shortcomings, such as reduced sensitivity to nuance, and may not fully capture the multidimensional nature of self-esteem [80]. Future research should consider more comprehensive assessments.

Third, the sampling strategy presents important constraints. The study used convenience-based snowball sampling, which, although practical given the absence of an accessible national registry, may have limited the representativeness of the findings and limited subgroup comparisons [81]. In addition, although the sample size met minimal statistical requirements, it remains relatively modest. The small population size of psychologists nationally (829 at the time of data collection) and feasibility constraints further limited recruitment, making the sample size relatively small. Consequently, subgroup comparisons, particularly across professional categories, marital status, or personal mental health history, among others, should be interpreted with caution due to small subgroup sizes [82]. For instance, the marked gender imbalance (87.9% female) reflects the female-majority of the field of psychology in Lebanon, but nevertheless restricts the extent to which findings can be generalized to male psychologists; such an imbalance limits statistical power to detect gender-specific effects. Also, participants were concentrated in certain subgroups, as over half were childless, ages clustered around 30–35, and more than 80% were clinical psychologists. These distributions constrain the ability to conduct meaningful subgroup comparisons and may obscure variability that exists within the broader psychologist population [82].

Fourth, several occupational factors known to influence psychologists’ mental health were not included in this study. Variables such as caseload intensity, exposure to vicarious or secondary trauma, access to supervision, burnout, and characteristics of the workplace environment were not assessed due to feasibility constraints and concerns about questionnaire length [83,84,85,86]. The omission of potentially relevant variables was inevitable given the pioneering nature of this research, but it may have led to a bias [87]. Such factors should be incorporated in future work to provide a more complete understanding of psychologists’ occupational well-being.

Finally, despite the study’s focus on mental health during a period of cumulative national crises, the survey did not directly assess contextual stress exposure related to Lebanon’s overlapping adversities, economic collapse, political instability, war-related threats, and the aftermath of the Beirut Port explosion. These crises often co-occur and interact, and each influences people differently based on their place of residence, direct or indirect exposure, ethnic background, and other factors, making it difficult to isolate or operationalize them without significantly extending the survey burden [58,88,89]. Because the primary focus was on clinicians’ psychological distress, crisis exposure variables were not included; however, future studies should examine these exposures separately or through dedicated instruments to clarify their unique and combined effects on psychologists’ well-being.

### 4.2. Healthcare and Clinical Implications

These findings underscore the need for contextually appropriate strategies to support the mental health of psychologists practicing within Lebanon’s prolonged crisis environment. In a system marked by institutional and economic constraints, feasible support should build on existing structures, particularly supervision, continuing education, and practice-analysis frameworks already recognized by the Lebanese Order of Psychologists (LOPsy). Creating calm, reflective spaces for individual or group supervision, including secure remote formats, and ensuring access to basic administrative and technological tools can help clinicians manage the emotional demands of their work [90].

Continuing education, which is required for license renewal in Lebanon, also offers a practical avenue to address emerging national challenges and crisis-related mental health needs.

However, because this study did not directly examine organizational conditions, caseload, or stressors specific to cumulative crises, further research tailored to the Lebanese context is needed to identify which structures and interventions would most effectively strengthen psychologists’ well-being.

## 5. Conclusions

This study offers an initial empirical overview of the mental health of psychologists practicing in Lebanon during a period of prolonged national instability. While the sample showed overall better psychological outcomes than those typically reported in the general population, a substantial proportion of psychologists nonetheless experienced meaningful levels of distress. Importantly, self-esteem emerged as a psychological resource, consistently linked with lower depression, anxiety, and stress, and with higher subjective well-being. This highlights the relevance of internal strengths in shaping clinicians’ capacity to cope within crisis settings.

Moreover, the study points to a critical public health implication: psychologists working in crisis-affected societies are themselves embedded in the same environment of instability, yet they are expected to serve as frontline providers for a population with growing mental health needs. Supporting their well-being is therefore not only a matter of individual care but a prerequisite for sustaining the mental health workforce during times of crises.

Beyond describing mental health status, the findings bring attention to potential vulnerable subgroups within the profession. Younger practitioners, those with personal psychological or health histories, individuals experiencing financial strain, and educational psychologists may constitute priority groups for targeted support.

Future research should build on these findings by incorporating richer assessments of occupational demands, contextual stressors, and crisis exposure to better understand the mechanisms underlying psychologists’ vulnerability and resilience. A deeper examination of these pathways will be essential for informing national policies and organizational interventions that protect the mental health workforce in Lebanon.

## Figures and Tables

**Table 1:** Sociodemographic and other characteristics of participants (N = 157).

	N (%)
**Age (years)**	
30–35	67 (42.7%)
36–41	27 (17.2%)
42–47	37 (23.6%)
48–53	26 (16.6%)
**Gender**	
Male	19 (12.1%)
Female	138 (87.9%)
**Education level**	
Master’s	133 (84.7%)
Doctorate	24 (15.3%)
**Marital status**	
Single, divorced, widowed	51 (32.5%)
Married	106 (67.5%)
**Having kids**	
No	81 (51.6%)
Yes	76 (48.4%)
**Health problems**	
No	131 (83.4%)
Yes	26 (16.6%)
**Psychological history**	
Nothing	111 (70.7%)
Anxiety disorders	26 (16.6%)
Eating disorders	3 (1.9%)
Mood disorders (depression, bipolar disorder)	11 (7.0%)
Obsessive–compulsive disorder	6 (3.8%)
**Working**	
No	6 (3.8%)
Yes	151 (96.2%)
**Governate**	
Beirut	23 (14.6%)
Mount Lebanon	81 (51.6%)
North Lebanon	20 (12.7%)
South Lebanon	8 (5.1%)
Bekaa	16 (10.2%)
Baalback	4 (2.5%)
Akkar	2 (1.3%)
Nabatiyeh	3 (1.9%)
**Monthly salary**	
<600 $	30 (19.1%)
601–900 $	24 (15.3%)
901–1200 $	27 (17.2%)
1201–1500 $	18 (11.5%)
1501–1800 $	14 (8.9%)
>1800 $	44 (28.0%)
**Work shift**	
Day shift	112 (71.3%)
Night shift	2 (1.3%)
Day/Night overlap	43 (27.4%)
**Number of years of experience**	
1–5 years	56 (35.7%)
6–10 years	52 (33.1%)
11–15 years	28 (17.8%)
16–20 years	12 (7.6%)
21–25 years	6 (3.8%)
>25 years	3 (1.9%)
**Profession**	
Clinical psychologist	129 (81.5%)
Educational psychologist	28 (17.8%)
**Psychotherapist**	
No	29 (18.5%)
Yes	128 (81.5%)
	**Mean ± SD**
**Self-Esteem**	3.94 ± 0.81
**Depression**	5.34 ± 5.24
**Anxiety**	6.77 ± 6.18
**Stress**	15.76 ± 6.33
**SWB**	13.61 ± 4.86
**ED**	5.48 ± 8.73

**Table 2:** Bivariate analysis of demographic factors associated with mental health (N = 157).

	Depression			Anxiety			Stress			SWB			EDs		
	Mean (SD)	*p*	Effect Size	Mean (SD)	*p*	Effect Size	Mean (SD)	*p*	Effect Size	Mean (SD)	*p*	Effect Size	Mean (SD)	*p*	Effect Size
**Age (years)**		0.141	0.041		**<0.001**	0.114		0.196	0.030		0.121	0.037		0.068	0.066
30–35 (n = 67)	0.72 (0.37)			0.83 (0.36)			16.84 (6.52)			12.85 (5.09)			0.76 (0.48)		
36–41 (n = 27)	0.66 (0.38)			0.74 (0.36)			15.89 (6.88)			12.93 (5.60)			0.63 (0.42)		
42–47 (n = 37)	0.64 (0.35)			0.70 (0.34)			15.05 (6.41)			15.03 (4.11)			0.45 (0.37)		
48–53 (n = 26)	0.51 (0.29)			0.48 (0.37)			13.88 (4.65)			14.27 (4.08)			0.63 (0.52)		
**Gender**		0.005	0.786		0.057	0.524		0.550	0.147		0.216	0.379		0.313	0.225
Male (n = 19)	0.91 (0.40)			0.90 (0.36)			19.95 (6.61)			12.00 (6.03)			0.55 (0.28)		
Female (n = 138)	0.63 (0.34)			0.71 (0.37)			15.88 (6.30)			13.83 (4.66)			0.66 (0.48)		
**Education level**		0.522	0.156		0.404	0.194		0.169	0.307		0.182	0.298		0.991	0.003
Master’s (n = 133)	0.67 (0.36)			0.74 (0.38)			16.06 (6.42)			13.39 (4.81)			0.65 (0.47)		
Doctorate (n = 24)	0.61 (0.39)			0.67 (0.33)			14.13 (5.63)			14.83 (5.07)			0.65 (0.49)		
**Marital status**		0.021	0.398		0.008	0.485		0.006	0.479		0.438	0.132		0.195	0.267
Single, divorced, widowed (n = 51)	0.75 (0.31)			0.85 (0.38)			17.76 (6.67)			13.18 (4.65)			0.73 (0.49)		
Married (n = 106)	0.61 (0.38)			0.67 (0.36)			14.80 (5.95)			13.82 (4.97)			0.61 (0.45)		
**Having kids**		0.018	0.420		0.028	0.373		0.123	0.247		0.135	0.240		0.050	0.384
No (n = 81)	0.73 (0.35)			0.79 (0.35)			16.52 (6.72)			13.05 (5.02)			0.74 (0.46)		
Yes (n = 76)	0.58 (0.36)			0.65 (0.39)			14.96 (5.82)			14.21 (4.64)			0.56 (0.47)		
**Health problems**		0.033	0.494		0.011	0.501		0.262	0.242		0.406	0.179		0.950	0.015
No (n = 131)	0.63 (0.36)			0.70 (0.38)			15.51 (6.23)			13.76 (4.93)			0.65 (0.47)		
Yes (n = 26)	0.81 (0.31)			0.88 (0.30)			17.04 (6.76)			12.88 (4.49)			0.66 (0.46)		
**Psychological history**		**0.002**	0.123		0.011	0.091		0.027	0.069		**<0.001**	0.129		0.060	0.084
Nothing (n = 111)	0.57 (0.34)			0.66 (0.35)			14.76 (6.11)			14.59 (4.52)			0.60 (0.45)		
Anxiety disorders (n = 26)	0.83 (0.36)			0.84 (0.42)			18.62 (6.05)			12.42 (5.18)			0.63 (0.45)		
Eating disorders (n = 3)	0.92 (0.15)			1.01 (0.09)			16.67 (1.15)			8.00 (1.73)			1.19 (0.30)		
Mood disorders (depression, bipolar disorder) (n = 11)	0.78 (0.31)			0.85 (0.45)			16.82 (8.10)			10.91 (4.74)			0.98 (0.58)		
Obsessive–compulsive disorder (n = 6)	0.91 (0.36)			1.04 (0.20)			19.67 (5.09)			8.50 (3.67)			0.60 (0.49)		
**Working**		0.774	0.131		0.985	0.008		0.495	0.285		0.842	0.083		0.845	0.115
No (n = 6)	0.62 (0.23)			0.73 (0.33)			17.50 (3.45)			14.00 (4.00)			0.70 (0.70)		
Yes (n = 151)	0.66 (0.36)			0.73 (0.38)			15.70 (6.41)			13.60 (4.90)			0.65 (0.47)		
**Monthly salary**		0.252	0.050		0.026	0.089		0.008	0.097		0.089	0.061		0.590	0.036
<600 $ (n = 30)	0.77 (0.31)			0.80 (0.35)			19.10 (5.07)			12.17 (4.71)			0.69 (0.51)		
601–900 $ (n = 24)	0.56 (0.42)			0.55 (0.42)			14.13 (7.64)			15.00 (4.99)			0.58 (0.49)		
901–1200 $ (n = 27)	0.72 (0.34)			0.85 (0.33)			16.26 (6.56)			13.85 (4.70)			0.78 (0.47)		
1201–1500 $ (n = 18)	0.61 (0.46)			0.75 (0.38)			15.67 (5.90)			11.56 (5.22)			0.75 (0.44)		
1501–1800 $ (n = 14)	0.52 (0.37)			0.52 (0.29)			12.07 (4.18)			15.00 (5.14)			0.54 (0.45)		
>1800 $ (n = 44)	0.67 (0.31)			0.76 (0.38)			15.30 (6.12)			14.09 (4.49)			0.59 (0.46)		
**Number of years of experience**		0.370	0.041		0.084	0.068		0.247	0.043		0.079	0.063		0.612	0.034
1–5 years (n = 56)	0.70 (0.36)			0.82 (0.41)			17.41 (6.54)			13.34 (4.73)			0.68 (0.49)		
6–10 years (n = 52)	0.67 (0.37)			0.65 (0.34)			15.04 (6.23)			13.15 (5.20)			0.67 (0.46)		
11–15 years (n = 28)	0.64 (0.34)			0.78 (0.30)			14.61 (5.40)			13.39 (4.91)			0.60 (0.45)		
16–20 years (n = 12)	0.68 (0.38)			0.61 (0.40)			15.33 (7.61)			15.75 (3.70)			0.51 (0.57)		
21–25 years (n = 6)	0.51 (0.15)			0.53 (0.32)			14.83 (5.67)			18.33 (1.97)			0.35 (0.26)		
>25 years (n = 3)	0.26 (0.45)			0.47 (0.58)			12.00 (5.29)			10.67 (3.51)			0.92 (0.57)		
**Profession**		0.006	0.615		0.109	0.340		0.005	0.594		0.108	0.337		0.199	0.342
Clinical psychologist (n = 129)	0.62 (0.35)			0.70 (0.38)			15.10 (6.15)			13.88 (4.76)			0.62 (0.46)		
Educational psychologist (n = 28)	0.84 (0.35)			0.83 (0.36)			18.79 (6.45)			12.25 (5.20)			0.78 (0.49)		
**Psychotherapist**		0.535	0.134		0.013	0.539		0.137	0.308		0.908	0.024		0.450	0.188
No (n = 29)	0.70 ± 0.38			0.89 (0.37)			17.34 (6.26)			13.52 (4.21)			0.58 (0.49)		
Yes (n = 128)	0.65 ± 0.36			0.69 (0.37)			15.41 (6.31)			13.63 (5.01)			0.67 (0.46)		

Note. Bold values indicate statistically significant correlations after Bonferroni–Holm correction for multiple comparisons, applied separately for each mental health outcome: depression (α = 0.002), anxiety (α = 0.001), stress (not significant), SWB (α = 0.001), and EDs (not significant).

**Table 3:** Pearson’s correlation between self-esteem and mental health outcomes (N = 157).

	Self-Esteem
Depression	−0.49 ***
Anxiety	−0.35 ***
Perceived stress	−0.48 ***
SWB	0.49 ***
Eds	0.01

*** *p* < 0.001.

**Table 4:** Multivariate linear regression taking self-esteem as the independent variable (N = 157).

	β	*p*	95% Confidence Interval	R^2^
Depression	−0.16	<0.001	−0.23; −0.09	0.449
Anxiety	−0.12	0.002	−0.20; −0.04	0.442
Stress	−2.98	<0.001	−4.08; −1.88	0.388
SWB	2.26	0.001	1.33; 3.18	0.449

## Data Availability

The data presented in this study are available on request from the corresponding author due to institutional ethical and legal reasons.

## Supplementary Materials

The following supporting information can be downloaded at https://www.mdpi.com/article/10.3390/healthcare14010080/s1. Figure S1: Subjective Well-Being (WHO-5) Q-Q Plot; Figure S2: Anxiety (LAS-10) Q-Q Plot; Figure S3: Perceived Stress (PSS-10) Q-Q Plot; Figure S4: Depression (PHQ-9) Q-Q Plot; Figure S5: Eating Disorders (EAT-26) Q-Q Plot; Figure S6: Self-esteem (A-SISE) Q-Q Plot; Table S1: Linear regression taking the depression as the dependent variable; Table S2: Linear regression taking anxiety as the dependent variable; Table S3: Linear regression taking the perceived stress as the dependent variable; Table S4: Linear regression taking the subjective well-being as the dependent variable.

## Abbreviations

The following abbreviations are used in this manuscript:

SWB	Subjective well-being
EDs	Eating disorders
PHQ-9	Patient Health Questionnaire Depression Scale
LAS-10	Lebanese Anxiety Scale
PSS-10	Perceived Stress Scale
EAT-26	The Eating Attitudes Test
A-SISE	The Arabic Single-Item Self-Esteem Scale
