# Supplementary material for: Mental Health of Psychologists During a Period of Cumulative Crises in Lebanon: The Predictive Role of Self-Esteem
Source: Healthcare (Basel). 2025 Dec 29;14(1):80. doi: 10.3390/healthcare14010080 (PMC12785700; doi:10.3390/healthcare14010080)
Supplement: Supplementary file 1 [file healthcare-14-00080-s001.zip › Supplementary Materials_Healthcare.pdf]

Supplementary Materials

Table S1. Linear regression taking the depression as the dependent variable ($R^2 = 0.449$)

	Unstandardized beta	Standardized beta	*p*	95% Confidence Interval
Marital status (married vs not married*)	-0.06	-0.08	0.382	-0.19 ; 0.07
Having kids (Yes vs no*)	-0.04	-0.05	0.631	-0.18 ; 0.11
Profession (Educational psychologist vs Clinical psychologist*)	0.15	0.17	**0.044**	0.004 ; 0.30
Psychological history (Anxiety disorders vs Nothing*)	0.12	0.13	0.112	-0.03 0.27
Psychological history (Eating disorders vs Nothing*)	0.42	0.17	**0.018**	0.07 ; 0.77
Psychological history (Mood disorders vs Nothing*)	0.17	0.13	0.088	-0.03 ; 0.36
Psychological history (Obsessive-compulsive disorder vs Nothing*)	0.12	0.07	0.384	-0.15 ; 0.38
Age (36-41 vs 30-35*)	-0.01	-0.01	0.893	-0.17; 0.15
Age (42-47 vs 30-35*)	-0.05	-0.06	0.490	-0.20 ; 0.10
Age (48-53 vs 30-35*)	-0.12	-0.11	0.205	-0.30 ; 0.06
Gender (female vs male*)	-0.16	-0.14	0.073	-0.33 ; 0.02
Health problems (Yes vs No*)	0.11	0.11	0.137	-0.04 ; 0.25
Work shift (Night vs Day shift*)	0.34	0.12	0.147	-0.12 ; 0.81
Work shift (Day/Night overlap vs Day shift*)	0.04	0.05	0.523	-0.08 ; 0.16
Self-esteem	-0.16	-0.36	**<0.001**	-0.23 ; -0.09

*Reference group. Bold numbers indicate significant p-value.

Table S2. Linear regression taking anxiety as the dependent variable ($R^2 = 0.442$)

	Unstandardized Beta	Standardized Beta	*p*	95% Confi-dence Interval
Marital status (married vs not married*)	-0.11	-0.14	0.106	-0.25 ; 0.02
Having kids (Yes vs no*)	0.06	0.08	0.409	-0.08 ; 0.21
Profession (Educational psychologist vs Clinical psychologist*)	0.04	0.04	0.616	-0.11 ; 0.19
Psychological history (Anxiety disorders vs Nothing*)	0.04	0.04	0.602	-0.12 ; 0.20
Psychological history (Eating disorders vs Nothing*)	0.46	0.18	**0.020**	0.07 ; 0.85
Psychological history (Mood disorders vs Nothing*)	0.18	0.12	0.096	-0.03 ; 0.40
Psychological history (Obsessive-compulsive disorder vs Nothing*)	0.22	0.12	0.141	-0.08 ; 0.52
Age (36-41 vs 30-35*)	-0.17	-0.17	0.067	-0.34 ; 0.01
Age (42-47 vs 30-35*)	-0.16	-0.18	0.057	-0.33 ; 0.01
Age (48-53 vs 30-35*)	-0.42	-0.41	**<0.001**	-0.63 ; -0.21
Gender (Female vs Male*)	-0.09	-0.07	0.338	-0.27 ; 0.10
Health problems (Yes vs No*)	0.10	0.10	0.185	-0.05 ; 0.25

Psychotherapist (Yes vs no*)	-0.13	-0.14	0.101	-0.29 ; 0.03
Monthly salary (601-900 vs <600$*)	-0.11	-0.10	0.287	-0.30 ; 0.09
Monthly salary (901-1200 vs <600$*)	0.03	0.03	0.723	-0.16 ; 0.22
Monthly salary (1201-1500 vs <600$*)	-0.02	-0.02	0.852	-0.23 ; 0.19
Monthly salary (1501-1800 vs <600$*)	-0.28	-0.20	**0.036**	-0.53 ; -0.02
Monthly salary (>1800 vs <600$*)	-0.01	-0.02	0.879	-0.19 ; 0.17
Number of years of experience (6-10 vs 1-5*)	-0.09	-0.11	0.221	-0.24 ; 0.06
Number of years of experience (11-15 vs 1-5*)	0.14	0.15	0.111	-0.03 ; 0.32
Number of years of experience (16-20 vs 1-5*)	-0.16	0.10	0.264	-0.12 ; 0.44
Number of years of experience (21-25 vs 1-5*)	-0.01	-0.01	0.949	-0.34 ; 0.32
Number of years of experience (> 25 vs 1-5*)	0.05	0.02	0.818	-0.39 ; 0.49
Self-esteem	-0.12	-0.26	**0.002**	-0.20 ; -0.04

*Reference group. Bold numbers indicate significant p-value.

Table S3. Linear regression taking the perceived stress as the dependent variable (R^2 = 0.388)

	**Unstandardized beta**	**Standardized beta**	***p***	**95% Confidence Interval**
Education level (Doctorate vs Masters*)	-1.08	-0.06	0.430	-3.69 ; 1.53
Marital status (married vs not married*)	-2.00	-0.15	0.087	-4.45 ; 0.45
Having kids (Yes vs no*)	0.74	0.06	0.543	-1.93 ; 3.41
Profession (Educational psychologist vs Clinical psychologist*)	2.43	0.15	0.063	0.10 ; 4.77
Psychotherapist (Yes vs no*)	0.30	0.02	0.828	-2.09 ; 2.69
Age (36-41 vs 30-35*)	-1.49	-0.09	0.294	-2.45 ; -0.53
Age (42-47 vs 30-35*)	-1.74	-0.12	0.204	-2.70 ; -0.78
Age (48-53 vs 30-35*)	-2.39	-0.14	0.160	-3.35 ; -1.43
Psychological history (Anxiety disorders vs Nothing*)	1.55	0.09	0.245	0.71 ; 2.39
Psychological history (Eating disorders vs Nothing*)	5.43	0.12	0.111	4.59 ; 6.27
Psychological history (Mood disorders vs Nothing*)	0.53	0.02	0.769	-0.31 ; 1.37
Psychological history (Obsessive-compulsive disorder vs Nothing*)	3.28	0.10	0.202	2.44 ; 4.12
Monthly salary (601-900 vs <600$*)	-2.48	-0.14	0.127	-2.97 ; -1.99
Monthly salary (901-1200 vs <600$*)	-2.63	-0.16	0.089	-3.12 ; -2.14
Monthly salary (1201-1500 vs <600$*)	-2.74	-0.14	0.122	-3.23 ; -2.25
Monthly salary (1501-1800 vs <600$*)	-5.98	-0.27	**0.003**	-6.47 ; -5.49
Monthly salary (>1800 vs <600$*)	-2.50	-0.18	0.092	-2.99 ; -2.01
Numbers of years of experience (6-10 vs 1-5 years*)	-1.31	-0.10	0.290	-1.80 ; -0.82
Numbers of years of experience (11-15 vs 1-5 years*)	-0.87	-0.05	0.556	-1.76 ; 0.02
Numbers of years of experience (16-20 vs 1-5 years*)	2.03	0.09	0.335	1.14 ; 2.92
Numbers of years of experience (21-25 vs 1-5 years*)	0.57	0.02	0.828	-0.32 ; 1.46

Numbers of years of experience (>25 vs 1-5 years*)	-2.53	-0.06	0.513	-3.42 ; -1.64
Self-esteem	-2.98	-0.38	**<0.001**	-4.08 ; -1.88

*Reference group. Bold numbers indicate significant p-value.

Table S4. Linear regression taking the subjective well-being as the dependent variable (R^2 = 0.449)

	**Unstandardized beta**	**Standardized beta**	***p***	**95% Confidence Interval**
Gender (female vs male*)	1.17	0.08	.277	-0.95; 3.29
Education level (Doctorate vs Masters*)	1.51	0.11	.139	-0.50; 3.51
Having kids (Yes vs no*)	-0.02	-0.002	.984	-1.61; 1.58
Profession (Educational psychologist vs Clinical psychologist*)	-0.49	-0.04	.601	-2.33; 1.35
Age (36-41 vs 30-35*)	-0.50	-0.04	.633	-2.57; 1.57
Age (42-47 vs 30-35*)	1.41	0.12	.164	-0.58; 3.41
Age (48-53 vs 30-35*)	0.37	0.03	.765	-2.09; 2.84
Psychological history (anxiety disorders vs nothing*)	-0.70	-0.05	.471	-2.63; 1.22
Psychological history (eating disorders vs nothing*)	-6.82	-0.19	**.007**	-11.73; -1.91
Psychological history (Mood disorders vs Nothing*)	-2.83	-0.15	**.032**	-5.42; -0.24
Psychological history (obsessive-compulsive disorder vs Nothing*)	-5.17	-0.21	**.007**	-8.90; -1.45
Monthly salary (601-900 vs <600$*)	1.92	0.14	.104	-0.40; 4.24
Monthly salary (901-1200 vs <600$*)	1.60	0.13	.153	-0.60; 3.81
Monthly salary (1201-1500 vs <600$*)	-0.03	-0.002	.981	-2.73; 2.66
Monthly salary (1501-1800 vs <600$*)	3.20	0.19	**.030**	0.31; 6.10
Monthly salary (>1800 vs <600$*)	1.60	0.15	.152	-0.60; 3.79
Work shift (Night vs Day shift*)	-5.56	-0.13	.094	-12.07; 0.96
Work shift (Day/Night overlap vs Day shift*)	-0.12	-0.01	.875	-1.68; 1.44
Numbers of years of experience (6-10 vs 1-5*)	-0.98	-0.10	.244	-2.65; 0.68
Numbers of years of experience (11-15 vs 1-5*)	-0.89	-0.07	.395	-2.95; 1.17
Numbers of years of experience (16-20 vs 1-5*)	0.31	0.02	.838	-2.71; 3.34
Numbers of years of experience (21-25 vs 1-5*)	2.44	0.10	.211	-1.40; 6.28
Numbers of years of experience (>25 vs 1-5*)	-3.08	-0.09	.289	-8.80; 2.65
Self-esteem	2.26	0.38	**0.001**	1.33; 3.18

*Reference group. Bold numbers indicate significant p-value.
