# Supplementary material for: Mental Health of Psychologists During a Period of Cumulative Crises in Lebanon: The Predictive Role of Self-Esteem
Source: Healthcare (Basel). 2025 Dec 29;14(1):80. doi: 10.3390/healthcare14010080 (PMC12785700; doi:10.3390/healthcare14010080)

Supplementary Materials

Figure S1. Subjective Well-Being (WHO-5) Q-Q Plot

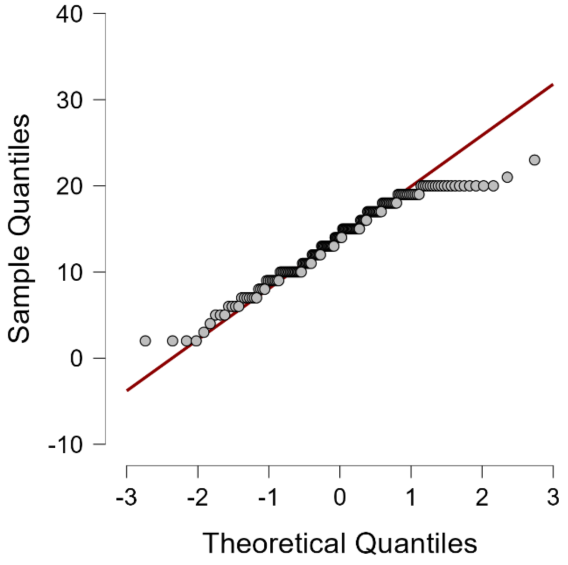

Figure S2. Anxiety (LAS-10) Q-Q Plot

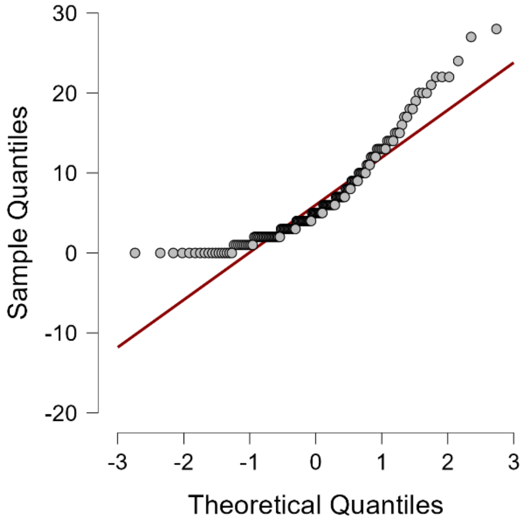

Figure S3. Perceived Stress (PSS-10) Q-Q Plot

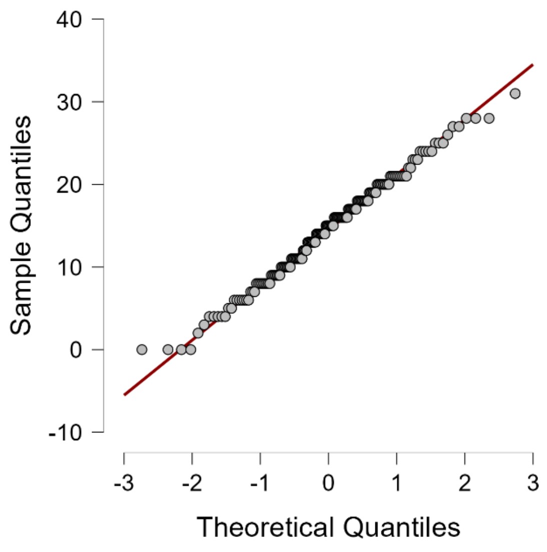

Figure S4. Depression (PHQ-9) Q-Q Plot

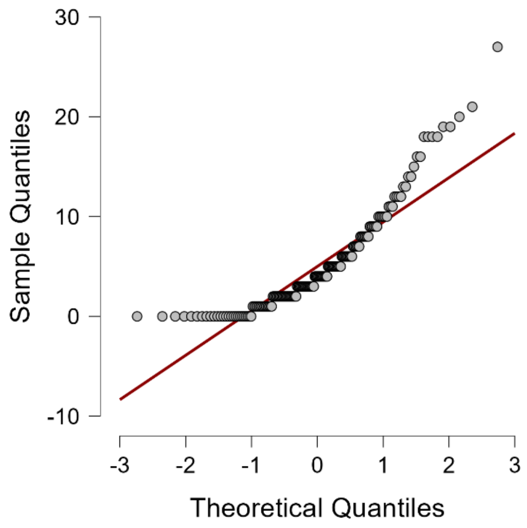

Figure S5. Eating Disorders (EAT-26) Q-Q Plot

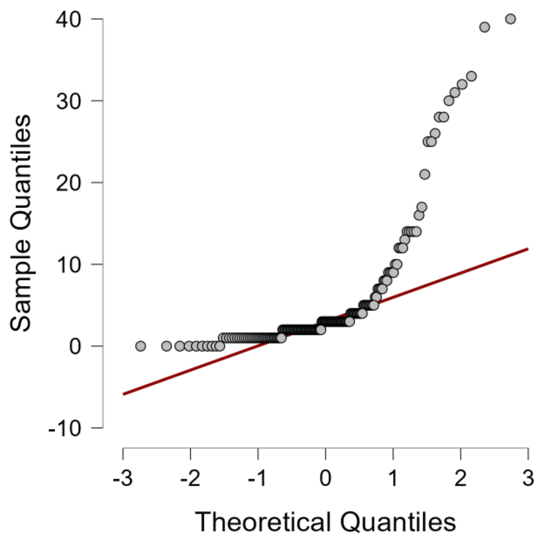

Figure S6. Self-esteem (A-SISE) Q-Q Plot

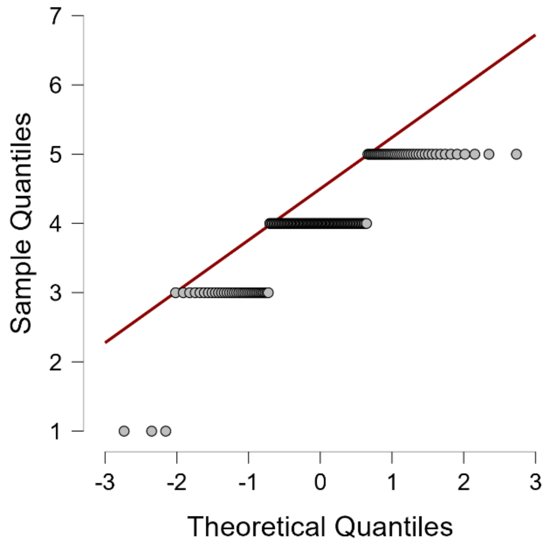

Supplement: Supplementary file 1 [file healthcare-14-00080-s001.zip › Supplementary Materials_QQ Plots.pdf]
